# Maternal exposure to cyclophosphamide during prepubertal life does not affect the cryosusceptibility of *in vitro* derived mouse embryos

**DOI:** 10.1530/RAF-25-0153

**Published:** 2026-01-29

**Authors:** Dhakshanya Predheepan, Sujith Raj Salian, Akshatha Daddangadi, Shubhashree Uppangala, Guruprasad Kalthur, Borut Kovačič, Satish Kumar Adiga

**Affiliations:** ^1^ Division of Clinical Embryology, Department of Reproductive Science, Kasturba Medical College, Manipal, Manipal Academy of Higher Education, Manipal, India; ^2^ Centre of Excellence in Clinical Embryology, Department of Reproductive Science, Kasturba Medical College, Manipal, Manipal Academy of Higher Education, Manipal, India; ^3^ Division of Reproductive Genetics, Department of Reproductive Science, Kasturba Medical College, Manipal Academy of Higher Education, Manipal, India; ^4^ Division of Reproductive Biology, Department of Reproductive Science, Kasturba Medical College, Manipal Academy of Higher Education, Manipal, India; ^5^ Laboratory of Reproductive Biology, Department of Reproductive Medicine and Gynaecological Endocrinology, University Medical Centre, Maribor, Slovenia; ^6^ Centre for Fertility Preservation, Department of Reproductive Science, Kasturba Medical College, Manipal, India

**Keywords:** cryopreservation, cyclophosphamide, embryo development, fertility, vitrification

## Abstract

**Abstract:**

Prepubertal exposure to gonadotoxic chemotherapy, such as cyclophosphamide (CY), poses a significant risk to long-term fertility by depleting ovarian reserve and impairing oocyte quality. Such treatments are commonly administered to young girls with cancer, and when these individuals later seek assisted reproductive technologies (ARTs), concerns arise regarding the developmental competence and cryotolerance of resulting embryos. Using a mouse model, this study evaluated the impact of prepubertal CY exposure (2 successive weekly doses of 75 mg/kg body weight at 2 weeks of age) on the cryosusceptibility of embryos derived via *in vitro* fertilization (IVF) at their reproductive phase. Oocytes were collected from superovulated females six weeks post-CY treatment and fertilized *in vitro*, and the resulting cleavage stage embryos were subjected to vitrification–warming. Embryo survival and quality were assessed by blastocyst formation, inner cell mass (ICM) proliferation *in vitro*, and the expression of key pluripotency markers (*Oct4*, *Sox2*, and *Nanog*). Results showed no significant differences between CY-exposed and control groups in terms of post-warming survival or marker expression. These findings suggest that maternal CY exposure during the prepubertal period does not adversely affect the cryo-resilience of IVF-derived embryos, offering reassurance for childhood cancer survivors seeking ART treatment.

**Lay summary:**

Chemotherapy given during childhood can harm future fertility, especially in girls who have not yet gone through puberty. One common chemotherapy drug, cyclophosphamide (CY), may reduce the number and quality of eggs in the ovaries. Cancer survivors may be more likely to need IVF (*in vitro* fertilization) or other fertility treatments, given the impact of the treatment on their fertility. In this study, a mouse model was used to understand whether early-life exposure to CY affects the ability of embryos (created through IVF) to survive freezing and thawing, a common part of IVF treatments. The study found that embryos from mice exposed to CY before puberty survived the freezing process just as well as those from unexposed mice. The quality and health of these embryos, measured by their development and important growth markers, were also similar. These findings provide hopeful news for young girls who receive certain types of chemotherapy, who may still have the option to use IVF in the future without increased risk of embryo damage during freezing.

## Introduction

The survival statistics for childhood cancer have significantly improved in recent decades due to breakthroughs in medical technology, treatment protocols, and increased awareness (Butler *et al.* 2021). Long-term follow-up studies have reported that female childhood cancer survivors confront an increased risk of experiencing diminished ovarian reserve, premature ovarian insufficiency, and infertility in adulthood (van Dorp *et al.* 2016, Anderson *et al.* 2018) primarily because of the alkylating agents used during chemotherapy (Spears *et al.* 2019, Hoefgen *et al.* 2023, Ida *et al.* 2023). Cyclophosphamide (CY) is a commonly employed alkylating chemotherapeutic drug in the fields of oncology and autoimmune disorders. CY specifically affects the prepubertal ovaries by causing loss of oogonia during prenatal development, as well as direct loss of primordial follicles and degeneration of follicles (Spears *et al.* 2019), thereby reducing the ability to conceive naturally. Since such cancer survivors frequently opt for assisted reproduction as a potential treatment strategy to overcome infertility, understanding the *in vitro* developmental competence of oocytes retrieved from diminished ovarian reserve due to CY therapy will provide significant insights to embryologists and clinicians.

Cryopreservation by vitrification has become an integral part of the assisted reproduction treatment strategy, especially for poor ovarian responders (Chi *et al.* 2015). Clinically, cryopreservation provides an additional safety for patients whose endometrium is not receptive to fresh embryo transfer (Shapiro *et al.* 2014). On the other hand, few studies have shown the adverse effects of vitrification on embryo and fetal development (Chen *et al.* 2024). Analysis of human and mouse placentas demonstrated that both frozen and fresh embryo transfer procedures significantly altered DNA methylation patterns, distinguishing them from those seen in placentas resulting from natural conception (Mani *et al.* 2022). In addition, there is a concern that the outcome of vitrification–warming is significantly affected when poor-quality embryos are cryopreserved (Liu *et al.* 2022). However, how embryos derived from oocytes exposed to a cytotoxic environment during the prepubertal life respond to cryopreservation has not been explored.

To address this in an experimental setting, we employed healthy prepubertal female Swiss albino mice, a well-established model for studying the reproductive effects of gonadotoxic agents (Salian *et al.* 2020, 2024) due to their physiological similarity to human ovarian development (Gaylord *et al.* 2025). Moreover, evaluating the embryological outcomes of prepubertal exposure to gonadotoxicity is not feasible in clinical cohorts, making the mouse model essential for generating insights that cannot be obtained through human studies. These female mice were exposed to CY treatment to assess the gonadotoxic effect on the follicular microenvironment and oocyte developmental potential during their reproductive phase. The oocytes were assessed for their fertilization rate, embryo development, susceptibility of embryos to cryopreservation by vitrification–warming, proliferation of the inner cell mass (ICM), and mRNA levels of pluripotency markers *Oct4*, *Sox2*, and *Nanog* in the blastocysts and ICM outgrowths.

## Materials and methods

### Animals and ethical clearance

Swiss albino mice were inbred and housed in the Central Animal Research Facility of the Manipal Academy of Higher Education. The 14-day-old mice obtained from timed pregnancies were housed with their mother until weaning at 4 weeks of age. Following weaning, the male and female mice were separated, and six mice per cage were housed under controlled conditions of 23 ± 2°C, 12 h light:12 h darkness cycle, and 50 ± 5% humidity and fed with a standard diet and water *ad libitum* (Predheepan *et al.* 2024*a*). Animal handling and research experiments were conducted as per the guidelines of the Institutional Animal Ethics Committee (IAEC/KMC/44/2021) and approved by the Manipal Academy of Higher Education. A minimum of three trials were performed for each outcome measure to ensure the reproducibility of the results.

### Experimental design

To induce the gonadotoxic effect induced by cancer therapy in an experimental setup, CY (Cat. No. C7397, Sigma-Aldrich, USA) was chosen due to its well-established ability to deplete the ovarian reserve. The dose selection was based on the blinded preliminary study, which compared dose regimes of CY administration to diminish ovarian reserve in the mouse model. A single CY dose of 50 and 75 mg/kg body weight (b.w.) was administered intraperitoneally on postnatal days 14 and 21 of healthy female mice. The control group received an equal amount of saline. These mice were regularly monitored for changes in body weight and loss of fur. While the 50 mg/kg dose did not result in a significant reduction in ovarian reserve compared to controls, the 75 mg/kg dose showed a significant reduction in ovarian reserve, validating its efficacy for our experimental model. Hence, the 75 mg/kg twice-dosing schedule was used for all subsequent experiments and ensured methodological consistency and reliability. All investigations were conducted 42 days following their last injection, corresponding to 63 days of the mice’s lifespan.

Female mice were superovulated and sacrificed (63 days of the mice’s lifespan) as described earlier (Predheepan *et al.* 2024*a*). The experimental groups involved in the study are control (C), CY treated (CY), control vitrified–warmed (C. VT), and CY treated vitrified–warmed (CY. VT). All experiments were carried out under identical culture conditions and by employing sibling oocytes for all the study groups. A minimum of six animals were involved per group per trial for the study.

### Assessment of ovarian histology and follicle count

A minimum of six ovaries were collected for each data point from animals across three study groups for the comparative assessment of ovarian histology in a blinded manner. Ovaries (either left or right) were weighed and fixed in Bouin’s fixative, dehydrated using increasing concentrations of ethanol, embedded in paraffin wax, and sectioned at 5 μm. The slides were deparaffinized in xylene, dehydrated in decreasing concentrations of ethyl alcohol, and stained with hematoxylin and eosin (Predheepan *et al.* 2022, Amonkar *et al.* 2023, Salian *et al.* 2024). The images were obtained using Olympus CX31 and DP23 imaging systems using CellSens Standard software 4.1.1 (build 26344) (Olympus Corporation, Japan) at 4X. In parallel, the ovarian reserve was quantitatively assessed by enzymatic ovarian digestion as described earlier (Nair *et al.* 2014). In brief, the ovaries were digested using 2 mg/mL trypsin–collagenase (Cat. No.: RM713, Himedia, India, and Cat. No.: 9001-12-1, Gibco, USA, respectively) dissolved in DMEM (Cat. No.: D5648, Sigma-Aldrich, USA) for 30 min. Isolated follicles from the ovaries were identified as primordial, primary, secondary, preantral, antral, and atretic follicles under light microscopy (Olympus IX 73, Olympus Corporation, Japan), as previously described (Amonkar *et al.* 2023).

### *In vitro* fertilization (IVF) and embryo culture

IVF was performed following a previously established protocol (Predheepan *et al.* 2024*a*). In brief, spermatozoa collected from the cauda epididymis of 10- to 12-week old male Swiss albino mice (*n* = 12) were released in pre-warmed Earl’s balanced salt solution (EBSS, Cat. No.: E2888, Sigma-Aldrich, USA) supplemented with 0.1% bovine serum albumin (BSA, Cat. No.: A3311, Sigma-Aldrich, USA). The sperm suspension was washed in potassium simplex optimization medium (KSOM-AA, Cat. No.: MR-107-D, Sigma-Aldrich, USA) supplemented with 0.1% BSA and then incubated for swim-up at 37°C for 45 min. The supernatant containing the motile spermatozoa was inseminated in a 4-well dish (Cat. No.: 176740, Thermo Fisher Scientific, USA) and overlaid with oil (Cat. No: V-OIL-P500; Vitromed, Germany). Cumulus oocyte complexes (COCs; *n* = 340, 522 for C and CY, respectively) collected from oviducts of female Swiss albino mice were washed and co-incubated with washed spermatozoa (2 × 10^6^ spermatozoa/mL) at 37°C with 5% CO_2_, 5% O_2_, and 89% N_2_ and assessed for fertilization at 10.5 h post-insemination (hpi). The normally fertilized oocytes were transferred to KSOM-AA supplemented with 0.1% BSA until 96 hpi, with embryonic development assessed at 24-h intervals using an inverted microscope (IX73, Olympus, Japan).

### Embryo vitrification and warming

Embryo vitrification–warming was performed using vitrification (Cat. No. VT601, Kitazato, Japan) and warming (Cat. No. VT602, Kitazato, Japan) kits with minor modifications in the protocol (Predheepan *et al.* 2024*a*). In brief, embryos at the 6- to 8-cell stage (56–60 hpi; *n* = 91, 144 for C and CY, respectively) were exposed to equilibration solution for 10–12 min and then to vitrification solution for 1 min, loaded onto a cryolock, plunged into liquid nitrogen, and stored for 1 h. These embryos were then warmed in a pre-warmed thawing solution for 1 min, followed by sequential transfers to dilution solution and washing solution for 3 and 6 min, respectively. Embryo survival was assessed after 3 h of culture.

### TUNEL assay

Apoptosis in blastocysts (*n* = 21, 30, 37, 34 for C, C.VT, CY, and CY.VT) was assessed by terminal deoxynucleotidyl transferase dUTP nick end labeling (TUNEL) assay (Cat. No. 12156792910, Roche, Germany) to detect DNA breaks indicative of early apoptosis (Predheepan *et al.* 2024*b*). At 108 hpi, the blastocysts were rinsed in phosphate-buffered saline (PBS), fixed overnight with 4% paraformaldehyde (w/v), and permeabilized in a phosphate buffer containing 0.1% sodium citrate (w/v), 0.5% BSA (w/v), and 0.5% Triton X-100 (v/v) for 1 h at room temperature. The embryos were washed and incubated with the TUNEL reaction mixture (9:1 ratio of TMR red labeling solution to enzyme solution) for 1 h at 37°C, followed by DAPI (4′,6′-diamidino-2-phenylindole) nuclear counterstain. The embryos were mounted on slides and examined under a fluorescence microscope (Carl Zeiss, Germany) to determine the apoptotic index, which was expressed as the percentage of TUNEL-positive cells per blastocyst.

### ICM outgrowth assay

The attachment and proliferation ability of the blastocysts developed *in vitro* (*n* = 33, 24, 48, 46 for C, C. VT, CY, and CY. VT, respectively) was assessed using the ICM outgrowth assay in a blinded manner as described earlier (D’Souza *et al.* 2018) with minor modifications. In brief, individual blastocysts at 108 hpi were transferred into 0.1% gelatin (Sigma-Aldrich, Cat. No. G1393) pre-coated multi-well dishes (Cat. No. 07-200-92, Thermo Fisher Scientific, USA) containing 200 μL DMEM (Sigma-Aldrich, Cat. No. D5648) supplemented with 20% fetal bovine serum (FBS, Cat. No. 10270106, Gibco, USA), overlaid with oil, and cultured. The ICM outgrowths (IO) at 204 hpi were imaged using an inverted microscope (Olympus IX 73, Japan) and were subjectively classified based on the relative size of the ICM expansion as reported previously (D’Souza *et al.* 2018): completely developed ICM outgrowth (CIO), large ICM outgrowth (LIO), small ICM outgrowth (SIO), and no ICM outgrowth (NIO).

### Isolation of total RNA, cDNA synthesis, and gene expression analysis

Total RNA was isolated from ≥ 60 blastocysts at 108 hpi or 10 CIO/LIO ICM outgrowths at 204 hpi per group per trial using the Ambion RNA extraction kit (Cat. No. 15596018, Thermo Fisher, USA). RNA (40 ng) from each sample was used for cDNA synthesis of all samples, regardless of the total RNA yield, ensuring uniformity across all the experimental groups. cDNA synthesis was performed using random primers using a high-capacity reverse transcription kit (Cat. No. 4368814, Applied Biosystems, USA). Quantitative polymerase chain reaction (qPCR) was carried out on a StepOneTM Real-Time PCR System using Taqman assays targeting *Oct4* (Mm0305391 – 7Ig1), *Sox2* (Mm0305 – 3810Is1), and *Nanog* (Mm020195 – 50Is1), with Actin b (*Actb, *Mm00607 – 939Is1) as the internal control. PCR amplification was conducted using a two-step procedure. The holding phase involved denaturation at 50°C for 2 min, followed by 95°C for 10 min. The cycling protocol comprised 40 cycles, each involving denaturation at 95°C for 15 s and annealing and extension at 60°C for 1 min. For relative gene expression, blastocysts that were not exposed to vitrification served as the reference sample for comparison at 108 hpi, while the ICM outgrowths of the CY-treated group were used as a reference sample at 204 hpi.

### Statistical analysis

Data are represented as mean ± standard error of the mean (SEM). Based on the distribution of data points, either one-way analysis of variance (ANOVA) or the Kruskal–Wallis test was used to analyze the data. All statistical tests were conducted using the GraphPad Instat software (GraphPad Inc., USA). GraphPad Prism 8 (GraphPad Prism software, USA) was used to provide the graphical representation of the data. The level of significance was set at 1% throughout the study to adopt a more stringent criterion and minimize the risk of false positives, given multiple comparisons in the present study. All the data provided are from a minimum of three independent trials.

## Results

**Figure 1:**
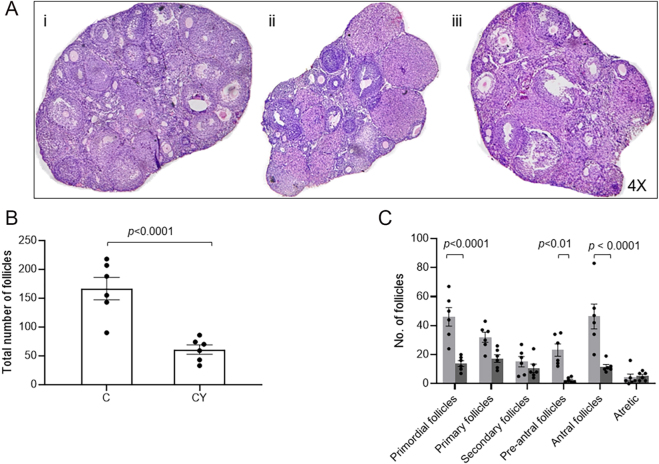
Effect of CY treatment on ovarian reserve. (A) Total follicle count of control mice, *n* = 6, and CY-treated mice, *n* = 6, where ‘*n*’ indicates the number of animals included in the analysis. (B) Ovarian follicle count of control mice (light gray bar), *n* = 6, and CY-treated mice (dark gray bar), *n *= 6. (C) Representative image of ovarian sections of (i) control and (ii–iii) CY-treated mice. Magnification: 4×.

The histological assessment showed a variation in the number of follicles between the control and CY groups (Fig. 1A). To validate this finding, the ovary was enzymatically digested to assess the follicle count, which revealed that there was a significant (*P* <0.0001) decline in the total number of follicles in the CY group compared to the control (Fig. 1B). In particular, the number of primordial follicles, preantral, and antral follicles in the CY group was significantly (*P* <0.01–0.0001) reduced when compared to the control group (Fig. 1C).

**Figure 2:**
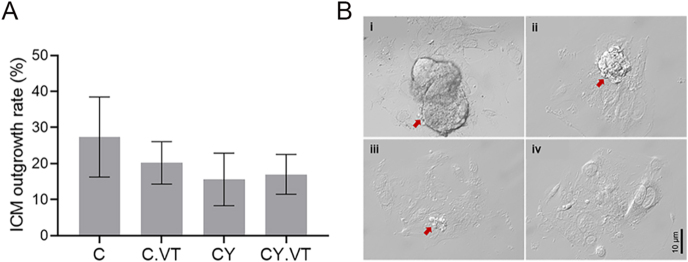
(A) Influence of vitrification–warming on complete ICM outgrowth rate, *n* = 33, 24, 48, 46 for C, C. VT, CY, and CY. VT, respectively, where ‘*n*’ indicates the number of blastocysts included in the analysis. (B) Representative phase-contrast images of (i) complete, (ii) large, (iii) small, and (iv) no ICM outgrowths (40×) at 204 hpi. The red arrows indicate the ICM outgrowths (scale bar: 10 μm).

The co-incubation of gametes revealed that the fertilization rate was comparable between the control (84.3 ± 6.8) and CY (88.3 ± 4.9) groups. When the resulting embryos were vitrified–warmed, the survival rate was comparable between the control (99.5 ± 0.5) and CY embryos (99 ± 1). Similarly, the blastocyst-forming ability of the vitrified–warmed CY embryos (71.6 ± 5.9) was comparable to that of the non-vitrified CY embryos (86.8 ± 3.2), control vitrified embryos (89.2 ± 2.8), and control non-vitrified embryos (88.9 ± 2.02). Moreover, the apoptotic index of the blastocysts was comparable between non-vitrified and vitrified groups in control (4.2 ± 0.7 vs 4.4 ± 0.4) and CY (4.1 ± 0.5 vs 4.2 ± 0.6) embryos, respectively. The ICM proliferation ability of blastocysts was assessed after 96 h of extended culture, which revealed that the ICM outgrowth rate was comparable between the vitrified and non-vitrified embryos in control and CY-treated groups (Fig. 2A).

**Figure 3:**
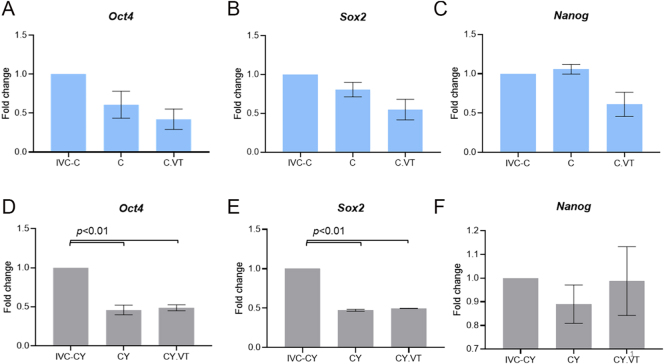
Effect of vitrification–warming on the mRNA level of *Oct4*, *Sox2*, and *Nanog* in control (A, B, C) and CY (D, E, F) blastocysts. *In vivo* derived control (IVC-C) blastocysts served as internal control for the untreated groups, and *in vivo* derived CY (IVC-CY) blastocysts served as internal control for the CY-treated groups. *Actb* served as a reference gene.

**Figure 4:**
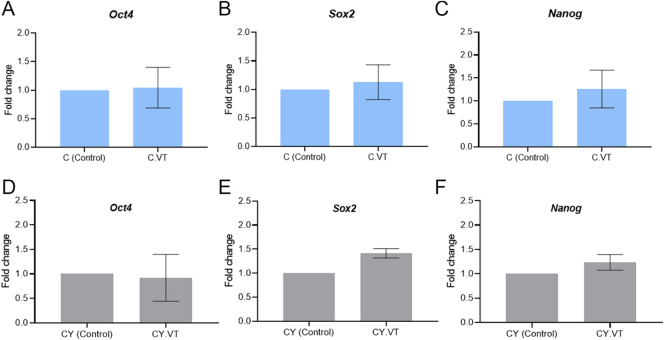
Effect of vitrification–warming on the mRNA level of *Oct4*, *Sox2*, and *Nanog* in control (A, B, C) and CY (D, E, F) ICM outgrowths. ICM outgrowths derived from non-vitrified embryos served as internal control for the respective experimental groups. *Actb* served as a reference gene.

Furthermore, the mRNA levels of pluripotency markers *Oct4*, *Sox2*, and *Nanog* were assessed in blastocysts and ICM outgrowths. The results showed that there was no significant difference in the level of transcripts observed between IVF-derived vitrified and non-vitrified embryos, as well as *in vivo* derived control embryos (Fig. 3A, B, C). Importantly, no substantial change in transcript levels was noted between IVF-derived vitrified and non-vitrified CY embryos, although a significant (*P* <0.01) difference in *Oct4* and *Sox2* transcript levels was observed when compared with *in vivo* derived CY embryos (Fig. 3D, E, F). *Oct4*, *Sox2*, and *Nanog* transcripts in ICM outgrowths were also comparable between the vitrified and non-vitrified control (Fig. 4A, B, C) and CY (Fig. 4D, E, F) embryos.

## Discussion

This study evaluated the impact of prepubertal CY exposure on the cryosusceptibility of embryos derived via IVF. Results showed no significant differences between CY-exposed and control groups in terms of post-warming survival or pluripotency.

The ovarian reserve is established before birth and gradually diminishes through controlled atresia until menopause (Laisk-Podar *et al.* 2016) or due to interventions detrimental to regular ovarian function. Chemotherapeutic drugs such as CY are recognized for dysregulation of folliculogenesis, leading to infertility (Spears *et al.* 2019). This disruption occurs through the depletion of primordial follicles, increased apoptosis, and the occurrence of follicular atresia (Donnez 2020). In accordance with this, our findings revealed that *in vivo* exposure of CY diminished the ovarian reserve, as indicated by the histological analysis and follicle count. Importantly, such gonadotoxic exposure compromises the ovarian environment, and subsequent procedures, such as *in vitro* culture and cryopreservation, can contribute to additional iatrogenic stress (Mao *et al.* 2022).

The results from the present study revealed that the *in vitro* fertilizing ability of *in vivo* derived oocytes exposed to CY was comparable to that of the control oocytes. Vitrification at the cleavage stage (day 3; 6–8 cells) remains a critical factor, particularly when fewer embryos are available for transfer, which is common in a clinical setting in patients with poor ovarian reserve, for example, childhood cancer survivors, with reference to the present experimental setting. Our findings suggested that the ability of CY embryos to survive cryopreservation and their developmental competence were comparable to those of the control embryos. Furthermore, the apoptotic index of blastocysts derived from oocytes exposed to CY and cryopreservation was comparable to that of the control blastocysts. Similarly, research has shown that the ability of oocytes exposed to CY to fertilize and develop embryos was comparable to that of the control (Koike *et al.* 2018). However, only the 6- to 8-cell stage embryos were exposed to cryopreservation by vitrification–warming, whereas the cryosusceptibility of other embryonic stages is unknown.

Pre-implantation embryos possess a highly effective system for responding to DNA damage following implantation, which plays a vital role in ensuring the development of the fetus (Jaroudi & SenGupta 2007). Studies have demonstrated increased apoptosis in the ICM section of the blastocysts developed *in vivo*, which might potentially lead to embryo death post-implantation due to an insufficient number of cells available to develop into different cell lineages (Adiga *et al.* 2010). Therefore, limiting the assessment of developmental potential to the blastocyst stage alone is not a dependable predictor of the possibility of successful implantation (Adiga *et al.* 2007, Kim *et al.* 2021). Henceforth, the developmental potential was assessed beyond the blastocyst stage. The results indicated that culturing the blastocysts derived from vitrified and non-vitrified groups of control and CY embryos had comparable ICM proliferation. Although the current study analyzed peri-implantation developmental competence using a surrogate marker for embryo implantation potential, this does not necessarily correlate with implantation potential and post-implantation outcomes. Hence, it is crucial for future research to examine the reproductive consequences and long-term risks associated with CY exposure, contributing to addressing the potential risk factors of CY treatment in cancer patients.

The regulation of early embryogenesis, cell fate decisions, and the ability to give rise to three germ layers (ectoderm, mesoderm, and endoderm) are governed by the pluripotency markers (Nichols & Smith 2009). Any perturbations in their expression may impair embryo viability and developmental competence (Zhang *et al.* 2013). Consequently, the mRNA levels of the well-established pluripotency markers *Oct4*, *Sox2*, and *Nanog* were examined. The gene expression analysis showed no discernible changes between vitrified and non-vitrified groups of the control and CY embryos in the blastocyst stage and ICM outgrowths, indicating that cryopreservation of embryos by vitrification may be a safe approach for poor ovarian responders. The downregulation observed in CY embryos developed *in vitro* compared to their *in vivo* counterparts may be attributed to the altered physiological conditions associated with the *in vitro* embryo culture systems. The strength of the study is that this is the first experimental investigation to analyze the developmental competence of embryos derived from oocytes exposed to a gonadotoxic follicular environment and their susceptibility to cryopreservation. Although this study used a mouse model due to ethical constraints, these findings hold significant translational importance. However, this study is constrained by its inability to assess the potential for *in vivo* implantation. Importantly, these intriguing findings are restricted to the gonadotoxicity resulting from CY pertaining to a particular dosage, not combination modalities, which are used in clinical practice to combat cancer effectively. Further direct dosage equivalence between mice and humans is not feasible due to differences in metabolism and pharmacokinetics. Therefore, it is crucial to understand the intricate genetic and epigenetic alterations of these therapies in the function and development of oocytes.

## Conclusion

These findings suggest that maternal CY exposure during the prepubertal period does not adversely affect the cryo-resilience of IVF-derived mouse embryos, offering reassurance to CY-exposed childhood cancer survivors seeking assisted reproductive technology treatment.

## Declaration of interest

The authors declare that there is no conflict of interest that could be perceived as prejudicing the impartiality of the work reported.

## Funding

DP is the recipient of a research fellowship from the University Grants Commission, Government of India. 

## Author contribution statement

SKA conceived and designed the experiments. DP, SRS, and ADP performed the experiments and acquired the data. DP and SU analyzed and interpreted the data. SKA and DP wrote the manuscript. SU, BK, and GK critically revised the manuscript for important intellectual content. DP has full access to all the data and takes responsibility for the accuracy of the data analysis. All authors have given final approval for publication.
